# Genome-Wide SNP Discovery in Indigenous Cattle Breeds of South Africa

**DOI:** 10.3389/fgene.2019.00273

**Published:** 2019-03-29

**Authors:** Avhashoni A. Zwane, Robert D. Schnabel, Jesse Hoff, Ananyo Choudhury, Mahlako Linah Makgahlela, Azwihangwisi Maiwashe, Este Van Marle-Koster, Jeremy F. Taylor

**Affiliations:** ^1^Department of Animal Breeding and Genetics, Agricultural Research Council-Animal Production, Irene, South Africa; ^2^Department of Animal and Wildlife Sciences, University of Pretoria, Pretoria, South Africa; ^3^Division of Animal Sciences, University of Missouri, Columbia, MO, United States; ^4^Informatics Institute, University of Missouri, Columbia, MO, United States; ^5^Sydney Brenner Institute of Molecular Bioscience, University of the Witwatersrand, Johannesburg, South Africa; ^6^Department of Animal, Wildlife and Grassland Sciences, University of the Free State, Bloemfontein, South Africa

**Keywords:** indigenous breeds, sequencing, novel variants, annotation, genes

## Abstract

Single nucleotide polymorphism arrays have created new possibilities for performing genome-wide studies to detect genomic regions harboring sequence variants that affect complex traits. However, the majority of validated SNPs for which allele frequencies have been estimated are limited primarily to European breeds. The objective of this study was to perform SNP discovery in three South African indigenous breeds (Afrikaner, Drakensberger, and Nguni) using whole genome sequencing. DNA was extracted from blood and hair samples, quantified and prepared at 50 ng/μl concentration for sequencing at the Agricultural Research Council Biotechnology Platform using an Illumina HiSeq 2500. The fastq files were used to call the variants using the Genome Analysis Tool Kit. A total of 1,678,360 were identified as novel using Run 6 of 1000 Bull Genomes Project. Annotation of the identified variants classified them into functional categories. Within the coding regions, about 30% of the SNPs were non-synonymous substitutions that encode for alternate amino acids. The study of distribution of SNP across the genome identified regions showing notable differences in the densities of SNPs among the breeds and highlighted many regions of functional significance. Gene ontology terms identified genes such as *MLANA*, *SYT10*, and *CDC42EP5* that have been associated with coat color in mouse, and *ADAMS3, DNAJC3*, and *PAG5* genes have been associated with fertility in cattle. Further analysis of the variants detected 688 candidate selective sweeps (ZH_p_ Z-scores ≤ -4) across all three breeds, of which 223 regions were assigned as being putative selective sweeps (ZH_p_ scores ≤-5). We also identified 96 regions with extremely low ZH_p_ Z-scores (≤-6) in Afrikaner and Nguni. Genes such as *KIT* and *MITF* that have been associated with skin pigmentation in cattle and *CACNA1C*, which has been associated with biopolar disorder in human, were identified in these regions. This study provides the first analysis of sequence data to discover SNPs in indigenous South African cattle breeds. The information will play an important role in our efforts to understand the genetic history of our cattle and in designing appropriate breed improvement programmes.

## Introduction

The development of next generation sequencing (NGS) technologies has enabled rapid and cost-effective generation of sequence data for SNP discovery in cattle (Le Roex et al., 2012; Mullen et al., 2012). These developments have also enabled the simultaneous estimation of SNP allele frequencies in a diverse range of reference populations (Van Tassell et al., 2008). Low and high-density SNP genotyping assays are available for performing genome-wide analyses in cattle (Matukumalli et al., 2009). However, while the available assays have been shown to be adequate for studies in European taurine breeds, they are less informative when applied to indicine or indigenous South African (SA) breeds (Gurgul et al., 2013; Zwane et al., 2016). Studies using the BovineSNP50 assay in indigenous SA breeds have shown substantially lower levels of linkage disequilibrium (LD) and lower minor allele frequencies (MAF) compared to those obtained in European taurine breeds (Edea et al., 2013; Makina et al., 2014). Furthermore, a study by Makina et al. (2015) using the BovineSNP50 assay for the detection of signatures of selection in indigenous SA breeds, also indicated reduced numbers of informative markers. Further analysis of these markers showed little evidence for the existence of breed-specific markers in indigenous SA cattle breeds (Zwane et al., 2016). Consequently, there is a limited utility for the implementation of these assays for genome-wide association studies (GWAS), quantitative trait locus (QTL) detection or for the identification of genes associated with economically important traits in indigenous SA breeds as observed by Albrechtsen et al. (2010).

South African indigenous cattle such as Afrikaner (AFR), Drakensberger (DRA), Nguni (NGI), Bonsmara, and Tuli have played a major role in traditional, social, and commercial history of the country (Scholtz, 2010). These breeds provide valuable farm animal genetic resources for beef production in combination with exotic beef breeds that were introduced many decades ago (Scholtz, 2010). The establishment of the SA indigenous cattle breeds and those from Africa was closely associated with human development and migration (Strydom, 2008). Africa’s indigenous cattle incorporate various crosses between Hamitic longhorn cattle (*Bos taurus*), zebu cattle (*Bos indicus*), and shorthorn cattle (Strydom, 2008). As the movement of man proceeded southward through Africa, new cattle breeds (zebu and sanga-types) were developed (Strydom, 2008). The zebu-type cattle include the Boran, Masai, and Sokoto breeds, while sanga-type (also known as *Bos taurus africanus*) includes the Afrikaner, Nguni, Pedi, Mashona, and Tuli (Hanotte et al., 2000; Strydom, 2008). Sanga cattle were introduced to SA during the migration of the San and Sudanic Bantu tribes to southern Africa and the arrival of Europeans during the fifteenth-century (Bachmann, 1983). Their substantial body mass and greater production in tsetse-free areas have made these breeds more appealing to the local farmers, which somewhat explains the abundance of these breeds and wide distribution throughout Africa (Mwai et al., 2015).

Indigenous breed of SA have made major contributions to livestock production because of their ability to adapt and produce in different SA production systems (Abin et al., 2016). These breeds have been participating in animal recording programmes with an average complete pedigree recording varying from 88.5% for the Nguni to 92.5% for the Afrikaner (Abin et al., 2016). The availability of the pedigree records have been essential for genetic evaluation using BLUP model in determining the selection efficiency and actual genetic change (Mostert, 2007; Groeneveld et al., 2009). However, crossbreeding and inbreeding within cattle breeds has been reported to have negative effects on production and fitness traits (Nazokkarmaher, 2016), and have contributed to loss of diversity in most cattle populations (Pienaar et al., 2014). The use of a small number of selected genotypes increases the chance of having undesirable recessive genes within a population, which may result in inbreeding depression in the near future (Abin et al., 2016). Quantitative breeding methods such as artificial insemination has resulted in more intense selection pressure on a number of traits of economic importance, which could have contributed to an increase in production efficiency. Therefore, maintaining within-breed genetic diversity is essential for selection (Oltenacu and Broom, 2010). Currently, relatively little information is available on SA cattle breeds at the genome level, including sequence variation. Therefore, sequencing the genomes of indigenous SA cattle could be beneficial in animal production, in understanding the genetic architecture of traits of economic importance, animal health and welfare, and in understanding the genetic basis of diseases, as well as genomic selection-based breeding program. Genome sequencing also presents opportunities for increased knowledge of the evolutionary histories of these breeds (Pool and Waddell, 2002).

NGS technologies have identified a large number of SNPs and insertions-deletions (Indels), with many variants remaining to be detected, especially in cattle breeds that are phylogenetically distinct from the more extensively studied European breeds (Choi et al., 2013). More than 60,000 putative SNPs were identified from the sequencing of reduced representation DNA libraries generated for 66 cattle from three populations (Van Tassell et al., 2008). More than 2 million novel SNPs were discovered from resequencing of a Fleckvieh bull (Eck et al., 2009). Furthermore, Kawahara-Miki et al. (2011) re-sequenced the genome of a single Kuchinoshima-Ushi (Japanese native cattle) bull and identified 6.3 million SNPs, of which more than 5.5 million (87%) were novel. Choi et al. (2014) reported a total of 10.4 million SNPs identified in Korean Hanwoo, Jeju Heugu and Holstein cattle, and found 54.12% novel SNPs and also detected 1,063,267 Indels in these genomes. This indicates that NGS technologies are effective for SNP discovery projects and can also be applied to variant discovery in indigenous SA cattle.

The use of sequence data for variant discovery and genotyping has the advantage of reduced SNP ascertainment bias compared to the use of commercially available SNP assays (Nielsen et al., 2011). SNP ascertainment bias influences the extent to which polymorphisms are shared across populations due to the distribution of allele frequencies within studied populations that may result in biases in measures of genetic differentiation, e.g., F_st_ estimates between populations affects the weighting of principal components, which in turn, can affect inferences about admixture in populations (McTavish and Hillis, 2015). Consequently, the sequencing of indigenous SA cattle genomes presents the potential to discover new SNPs for inclusion in existing SNP assays or for developing custom-made SNP chips for local SA populations. This information can also improve the accuracy of inferences made in population studies and the genome-wide detection of genes associated with complex traits such as disease resistance (Pool et al., 2010). It also holds potential for the identification of breed informative SNPs for breed assignment in SA populations (Ramos et al., 2011).

Fewer studies were done to understand the genetic variation of indigenous SA breeds at the genome level. Studies have focussed on the use of microsatellite markers and available bovine SNP assays to determine the extent of genetic diversity among Nguni, Bonsmara, Drakensberger, and Afrikaner cattle (Makina et al., 2014; Pienaar, 2014; Sanarana et al., 2016). Other studies include the identification of genes for tick resistance and copy number variation (CNV) in Nguni, as well as determining the extent of LD in SA cattle as compared to the European taurine breeds (Makina et al., 2015; Mapholi, 2015; Wang et al., 2015). These studies have provided a basis for understanding the genetic diversity and variation among these cattle breeds. To date, limited sequence data have been generated for indigenous SA cattle breeds. Breeds such as Brahman, Afrikaner and Tuli (African indicine), representing Australian populations, have been sequenced and analyzed resulting in 3.56 million new SNPs being submitted to dbSNP (Barris et al., 2012). The objective of this study was to search for novel SNPs the three indigenous SA cattle breeds [i.e., Afrikaner (AFR), Drakensberger (DRA), and Nguni (NGI)] using whole genome sequencing, and use identified SNPs perform functional enrichment analysis.

## Materials and Methods

### Pedigree Analysis and Sample Identification

The available pedigree data for each breed were obtained from the Agricultural Research Council (ARC) Integrated Registration and Genetic Information System (INTERGIS) database. Pedigree analysis of Afrikaner (*n* = 251,964), Drakensberger (*n* = 198,237), and Nguni (*n* = 241,491) were performed within breed to identify the least related individuals in these populations. Relationship coefficients between individuals were estimated using the method of Meuwissen and Luo (1992) implemented in the PEDIG software (Boichard, 2002), where males born between 2006 and 2012 were considered to be the reference population. In total, 90 least related animals across breeds (i.e., 30 animals per breed) with average relationship coefficients of 0.006, 0.008, and 0.0008 for Afrikaner, Drakensberger, and Nguni, respectively were selected across all nine SA provinces for sequencing to span the cattle’s genetic diversity, and breeder’s consent was obtained from the animal owners.

### Sample Collection, Library Construction, and DNA Sequencing

Sampling of blood and hair was performed with the approval of the Animal Ethics Committee of the University of Pretoria (EC: S4285-15), according to guidelines for the proper handling of animals during sample collection. Genomic DNA was extracted from whole blood (200 μl/sample) using the Roche DNA extraction Kit (Roche, Germany) following the standard protocol of the manufacturer. The procedure included a proteinase K digestion followed by column purification for the extraction of high quality DNA. The extraction of DNA from hair roots was performed using an optimized Phenol-Chloroform protocol (Sambrook and Russell, 2006), that included a Proteinase K and Dithiothreitol digestion followed by phenol-chloroform extraction and centrifugal dialysis with Centricon concentrators (Slikas et al., 2000). The quality of the extracted DNA samples was assessed using a NanoDrop UV/Vis Spectrophotometer (NanoDrop ND-1000) and verified using a Qubit^®^ 2.0 Fluorometer (Thermo Fisher Scientific). All DNA samples were maintained at a concentration of 50 ng/μl in preparation for NGS sequencing at the ARC-Biotechnology Platform.

Equimolar DNA pools were prepared for each breed using 170 ng of DNA per animal, and each DNA pool contained 30 animals per breed. Genomic libraries were prepared with the Truseq DNA sample preparation kit v2 (Illumina, San Diego, CA, United States) using 1 μg of genomic DNA according to the manufacturer’s instructions. DNA was fragmented using a Covaris E220 sonicator (350 bp), end-repaired and A-tailed followed by the ligation of adapters (TruSeq, Illumina) and 12 cycles of polymerase chain reaction (PCR) were performed. Quantities and the quality of usable material for each of the libraries were estimated by qPCR (KAPA Library Quantification Kit–Illumina Genome Analyzer-SYBR Fast Universal). The automated cBot Cluster Generation System (Illumina, San Diego, CA, United States) was used to generate clusters on the flow cell. Each DNA pool was then sequenced (paired-end; read length 125 bp) on a single lane of the Illumina HiSeq 2500. The resulting images were analyzed with the Bcl2fastq v2.0 (Illumina) to generate the raw fastq files (Ramos et al., 2009; Van et al., 2013; Boutet et al., 2016).

Sequence reads were filtered for base quality and adapter trimming using Trimmomatic v0.33 (Bolger et al., 2014). Reads were trimmed if four consecutive bases had an average Phred-like quality score of less than 20. After trimming, only pairs of DNA sequences for which each read exceeded 35 bp were retained for analysis. Sequence reads were aligned to the UMD3.1 reference genome using the Burrows-Wheeler aligner (BWA-MEM) v0.7, a software package for mapping lowly-divergent sequences against a large reference genome (Li and Durbin, 2009). The alignments were coordinate sorted and converted to the BAM format using SAMtools v1.2 (Ramirez-Gonzalez et al., 2012). Data were then formatted for variant calling using Picard v1.135, by marking duplicate reads (Li et al., 2009).

### Variant Discovery and SNP Annotation

Variant discovery was performed within breed according to GATK Best Practices using the genomic variant call format (GVCF) workflow, using HaplotypeCaller (Van der Auwera et al., 2013). The workflow includes data pre-processing steps and calling variants separately for each population using a command that is specific for paired-end data. The pre-processing steps include realigner target creator to generate intervals for each chromosome for Indel realignment, depth of coverage estimation for each chromosome, base recalibration (using dbSNP build 143 as known variants), analyzing covariates/variables and printing reads. Genotype calling was performed separately for each chromosome to generate GVCF files for variant calling, using –sample ploidy of 60 for pooled samples. The workflow included a joint analysis step that empowers variant discovery by providing the ability to leverage population-wide information from a cohort of samples (in this case three populations), allowing the detection of variants with greater sensitivity and genotyping samples as accurately as possible (GATK Best Practices; Bareke et al., 2013). Variants were generated in VCF files, and the genotypes were called for each breed with a minimum genotype quality of 20 (Aslam et al., 2012).

To reduce the false discovery rate, hard filtering steps were conducted using the following criteria: Phred scaled polymorphism probability (QUAL) < 30.0, variant confidence normalized by depth (QD) < 2.0, mapping quality (MQ) < 40.0, strand bias (FS) > 60.0, HaplotypeScore > 13.0, MQRankSum < -12.5, and ReadPosRank-Sum < -8.0 (GATK Best Practices; Choi et al., 2015). All SNPs that passed these criteria were consequently categorized into fixed (homozygous non-reference assembly nucleotide genotypes within the breed) or segregating (variable/heterozygous genotypes identified in the breed) (Aslam et al., 2012). The transition-to-transversion (Ti/Tv) ratio for each SNP call was calculated for each population as an indicator of potential sequencing errors (Choi et al., 2015) using VCFtools (Danecek et al., 2011). This is the ratio of the number of transitions (interchanges of either purines, A < - > G or pyrimidines, C < - > T) to the number of transversions (interchanges of purine for pyrimidine bases), for a pair of DNA sequences (Mitchell, 2015).

SNP annotation and the functional consequences of sequence variants were predicted using the Variant Effect Predictor (VEP) v2.0 tool, Ensembl Build 87 (Huang et al., 2009; McLaren et al., 2010). For all input variants, VEP provides detailed annotations for transcripts, proteins, and regulatory regions, and also provides phenotype information for known variants (McLaren et al., 2016). The functional effects of each SNP were estimated, and all SNPs were assigned with a diverse range of functional categories based on genomic coordinates, functional class, codon change, gene name, transcript biotype, gene coding, transcript ID, exon rank, and corresponding genotype (Choi et al., 2015). Annotation results were downloaded for further downstream analysis. The identified variants were verified by using data from European taurine or indicine breeds that were available from Run 6 of the 1000 Bull Genomes Project, consisting of more than 2,700 cattle and 86,474,165 million variants (Frischknecht et al., 2017) to identify novel SNPs present within the SA breeds.

### Assessment of SNP Density

SNPs were examined to determine their distribution throughout the genome, identify regions enriched for novel and non-synonymous SNPs and identify genes associated with enriched regions. To identify genomic regions of exceptional SNP densities, we compared the distribution of four different categories of SNPs [all SNPs, missense SNPs, LoF SNPs (stop gain and stop loss) and novel SNPs] in 1 Mb non-overlapping windows across the genome in each breed. The choice of window was based on a recent publication by Das et al. (2015). An in-house script was used to compute the SNP densities and R-script was used to visualize the distribution (Turner, 2014). The top 1% windows for each breed and category were annotated with the Ensembl Cow database, Release 87^1^ and when one or more genes were found in a window, the corresponding windows were ascribed the gene names.

### Identification of Selective Sweeps

Identification of selective sweeps was performed using the approach of Rubin et al. (2012) that makes provision for the identification of variants from pooled whole genome sequence data. This method determines, for each pool and SNP, the numbers of reads corresponding to the most (*n*_MAJ_) and least abundant alleles (*n*_MIN_) and for each window in each breed pool, a pooled heterozygosity score is calculated as:

$${\text{H}}_{\text{p}}=2\sum n_{\text{MAJ}}\sum n_{\text{MIN}}/{\left(\sum n_{\text{MAJ}}+\sum n_{\text{MIN}}\right)}^{2}$$

where Σ*n*_MAJ_ and Σ*n*_MIN_ are the sums of *n*_MAJ_ and *n*_MIN_ for all SNPs in the window. Individual H_p_ values are then Z-transformed as follows:

$${\text{ZH}}_{\text{p}}=(H_{\text{p}}-\mu H_{\text{p}})/\sigma H_{\text{p}}$$

where *μH_p_* and *σH_p_* are the mean and standard deviation for the H_p_ scores. To detect putative selective sweeps, a whole genome screen was performed to identify genomic regions with an excess of homozygosity (heterozygote deficiency) from the autosomes. SNPs were used to calculate Z-transformations of the pooled heterozygosity (ZH_p_) in each of the three breeds, and the number of sequence reads containing major and minor alleles were also counted. Subsequently, a 50% overlapping sliding window approach with 150 kb windows was used to compute ZH_p_ in each of the windows, and plot the distribution of SNP counts within these windows. Windows with ZH_p_ Z-scores of ≤-4 were retained as candidate selective sweep regions and regions with ZHp Z-scores of ≤-5 as putative selective sweeps. In addition, animal QTLdb was used to retrieve quantitative trait loci (QTL) information and visualize the QTL located within the putative selective sweep regions (Hu et al., 2013).

## Results

### Sequencing and Mapping

Sequencing of AFR, DRA, and NGI generated approximately 1.8 billion (184 Gb) of high quality paired-end reads using an Illumina HiSeq 2500 sequencer, of which 99% of the reads were mapped to the bovine reference genome (UMD 3.1). PCR duplicates were marked and reads were realigned around insertion and deletion events resulting in approximately 1.7 billion sequence reads (90.2%) across the three breeds, with an average coverage of 21.1-fold across the reference genome (Table 1). The Ti/Tv ratio and heterozygous/homozygous variant ratios are computed in genetic studies as a quality control measure for sequence data. To evaluate the quality of the detected SNPs, the Ti/Tv ratio was computed and found to be similar for each breed (AFR:2.20, DRA:2.23, NGI:2.22).

**Table 1:** Sequencing results for indigenous Afrikaner, Drakensberger, and Nguni cattle breeds.

Breed	Animals pooled	Raw reads	Non-duplicated reads	Properly paired reads	Mapped reads	High quality mapped reads	Average coverage
AFR	30	537,681,018	518,717,587	500,986,036	536,215,468	424,043,570 (79%)	21.2X
DRA	30	540,797,394	498,063,449	502,707,076	537,486,252	385,388,748 (71%)	15.4X
NGI	30	682,407,201	646,078,421	640,580,750	680,935,451	528,151,411 (77%)	26.6.X
Total	90	1,760,885,613	1,662,859,457	1,644,273,862	1,754,637,171	1,337,583,729 (76%)	21.1X

### Variant Detection

**FIGURE 1:**
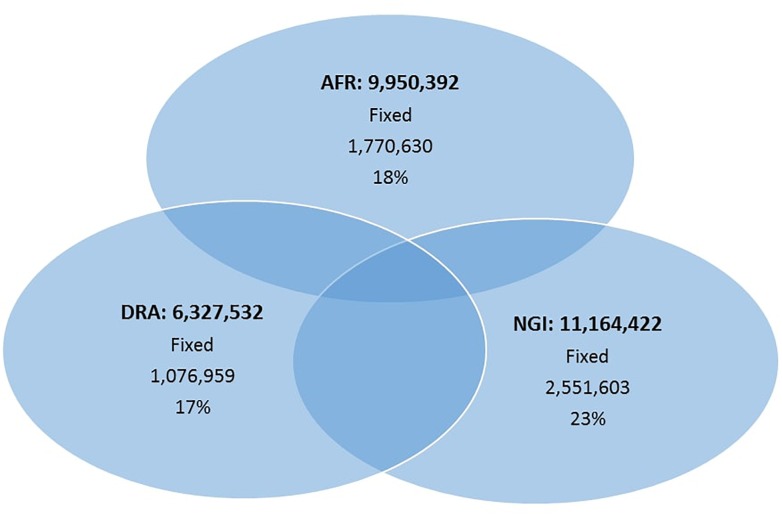
The number of SNPs identified among the three indigenous South African breeds.

A total of 17.6 million variants were identified in the three studied breeds with the greatest number of variants in NGI and AFR and lowest in DRA (Table 2). The detected variants comprised 89% SNPs and 11% Indels. From the total number of identified SNPs, on average, 58% of the SNPs were shared among the three cattle populations (Figure 1) with the highest number of SNPs shared between AFR and NGI.

**Table 2:** Summary of SNPs and Indels identified in Afrikaner (AFR), Drakensberger (DRA), and Nguni (NGI).

		SNPs		Indels	
Breed	No. variants	No. SNPs	Proportion SNPs	No. Indels	Proportion indels
AFR	11,165,172	9,950,384	0.89	1,212,231	0.11
DRA	7,049,789	6,327,515	0.90	721,628	0.10
NGI	12,514,952	11,164,415	0.89	1,347,215	0.11
Total	17,243,304	15,442, 314	0.89	1,908,137	0.11

### Validation of SNPs Using 1000 Bull Genomes Data

**FIGURE 2:**
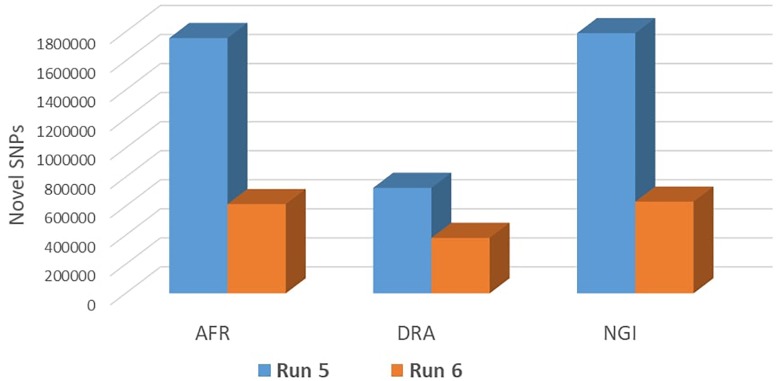
Validation of novel SNPs using Run 6 of 1000 Genomes Project data.

Run 6 of the 1000 Bull Genomes Project (Daetwyler et al., 2014; Frischknecht et al., 2017) was used to identify SNPs in the three SA breeds that are in common with other cattle breeds worldwide (i.e., taurine and indicine). On average, 93% of all SNPs identified in the three SA indigenous breeds were also shared among the breeds represented in the 1000 Bull Genomes Project data (Table 3). The remaining 7% of SNPs appear to be unique to SA indigenous breeds, AFR (7%), DRA (6%), and NGI (7%), using Run 6 of 1000 Bull Genomes Project data (Figure 2).

**Table 3:** Novel variants identified in the three breeds through comparison to 1000 Bull Genomes Project Run 6 data.

	All Variants				SNPs			
Breed	Known variants	Novel variants	Total	Proportion novel variants	Known SNPs	Novel SNPs	Total	Proportion novel SNPs
AFR	9,381,545	614,536	9,996,081	0.07	8,576,732	617,296	9,194,028	0.07
DRA	6,307,154	381,743	6,688,897	0.06	5,764,627	413,795	6,178,422	0.06
NGI	10,693,999	631,412	11,325,411	0.07	9,793,635	647,269	10,440,904	0.07
Total	26,382,698	1,627,691	28,010,389	0.07 (Av)	24,134,994	1,678,360	25,813,354	0.07 (Av)

### SNP Annotation and Analysis of Functional Enrichment

SNP annotation using VEP Ensembl gene annotation and dbSNP indicated that 62% of the SNPs were located in intergenic regions (AFR:62%, DRA:61%, NGI:62%), 29% were located in genic regions including introns, splice sites, and exons. Fewer SNPs (9%) were located in upstream/downstream regions (i.e., 5′ and 3′ untranslated regions; UTR). Tables 4, 5 indicate the distribution of SNPs and Indels detected within each functional class within genic regions.

**Table 4:** Counts of SNPs within each functional class for gene regions.

SNP class			Count				Total
	AFR	%	DRA	%	NGI	%	
Downstream_gene	437,355	4.4	288,515	4.6	440,357	3.9	1,166,227
Stop_lost	318	0.003	200	0.003	350	0.003	868
Stop_gain	38	0.0004	22	0.0003	15	0.0001	75
Splice_site	7,650	0.08	5,305	0.008	7,553	0.07	20,508
Upstream_gene	433,495	4.4	435,935	6.9	435,955	3.9	1,305,385
Intronic	2,726,502	27.4	1,800,155	28.4	2,731,530	24.5	7,258,187
miRNA	32,911	0.33	21,670	0.34	33,544	0.3	88,125
Synonymous_coding	38,537	0.4	29,836	0.47	40,694	0.36	109,067
Nonsynonymous_coding	31,205	0.31	22,395	0.35	31,130	0.28	84,730
3′_UTR	18,999	0.2	13,163	0.21	18,968	1.7	51,130
5′_UTR	3,974	0.04	3,055	0.05	3,805	0.034	10,834
Within_non_coding_gene	8,561	0.09	5,608	0.09	8,725	0.08	22,894
Essential_splice_site	182	0.002	124	0.002	192	0.002	498
Total	3,739,545	37.6	2,625,859	41.5	3,752,626	33.6	10,033,300

**Table 5:** Counts of Indels by functional class for gene regions.

Indel class		Count					Total
	AFR	%	DRA	%	NGI	%	
Downstream_gene	126,159	10.4	73,669	10.2	50,823	3.8	250,651
Stop_lost	43	0.004	49	0.007	26	0.002	118
Stop_gain	82	0.007	115	0.016	34	0.003	231
Splice_site	2481	0.2	1667	0.23	952	0.007	5,100
Upstream_gene	123,341	10.2	71,747	10.4	48,080	3.6	243,168
Intronic	745,500	61.5	431,225	59.8	317,114	23.5	1,493,839
miRNA	10,296	0.85	5,644	0.8	3,816	0.28	19,756
Synonymous_coding	1,004	0.08	855	0.12	449	0.33	2,308
Non-synonymous_coding	2,943	0.24	2,293	0.32	1,145	0.008	6,381
3′_UTR	5,574	0.46	3,165	0.44	2,166	0.16	10,905
5 ′_UTR	842	0.07	660	0.01	376	0.028	1,878
Within_non_coding_gene	2,141	0.18	1,311	0.18	545	0.04	3,997
Total	1,020,406	84.1	592,400	82.1	425,526	31.6	2,038,332

Of the total number of Indels, 61% were located in intergenic regions, 28% in genic regions including introns, exons and splice sites, and 1% were located in untranlsted regions. In AFR, there were 433,495 (4.4%) SNPs located within 5 kb upstream of a transcription start site and 437,355 (4.4%) SNPs within 5 kb downstream of a transcription stop site; 3,974 (0.04%) SNPs were located in a 5′UTR and 18,999 (0.2%) in a 3′UTR. These totals were slightly different in other two breeds, and were slightly lower in NGI. A total of 20,508 SNPs across the three breeds were located in splice sites, and 498 SNPs were in splice/donor sites. A total of 109,067 non-synonymous single nucleotide polymorphisms (nsSNP) substitutions were observed. There were 868 SNPs predicted to cause premature stop codons and 75 to cause gains in coding sequence across the breeds.

### Distribution of SNPs and Their Associated Genes

**FIGURE 3:**
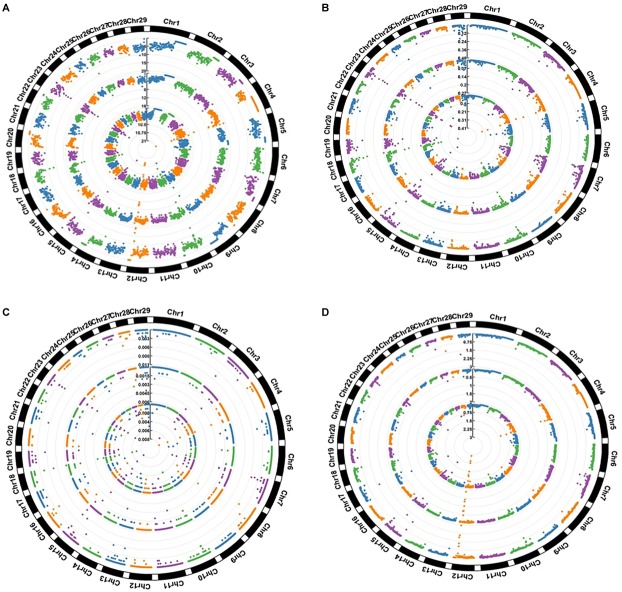
Distribution of SNPs in 1 Mb windows **(A)** All SNPs **(B)** Missense SNPs **(C)** LoF (stop gain and stop loss) SNPs **(D)** Novel SNPs. The values expressed are Number of SNPs per kilobase. The inner circle represents AFR, the middle circle represent DRA and the outer circle represent NGI.

Figure 3 summarizes the distribution of four different categories of SNPs in the three breeds. The figure shows that while most of regions found to be enriched for these four categories were shared, some were breed specific. For example, regions spanning multiple 1 Mb windows in chr 12, chr 18, chr 23 were found to show high overall SNP density in all three breeds, while a window on chr 28 (44,000,000–45,000,000) appeared among the top 1% regions in AFR and NG but did not appear even in the top 10% in DRA. Similar trends were also observed in the distribution of missense variants where regions on chr 4, chr 10, chr 15, chr 18, chr 19, chr 23, chr 25, and chr 29 were observed to be enriched with these variants in all three of the breeds. On the other hand two consecutive windows chr 5 (containing *ATN1*, *C3AR1*, and other genes) were found to be among top 1% missense variants in AFR but were not observed among the top 10% regions for either NGI or DRA.

Many of the regions which showed top 1% SNP densities in one or more of the categories have been found to be associated to disease or critical functional processes in previous studies, highlighting possible evolutionary rationale for their unusual densities. A window on Chr 4 corresponding to the *TRB* genes (*TRBV15, 16, 29, 30, and TRBV6*) was found to be in the top 1% both for overall SNP density as well as missense SNPs density in AFR and DRA (but not in NGI). *TRB* genes have been associated with domestication in cattle, showing strong evolutionary pressure (Connelley et al., 2009). The *CLEC5A* gene also from the same region of Chr 4 has been associated with diseases (increased level of swine influenza) in pigs (Fraser, 2018). *CDC42EP5* gene on Chr 18 (region detected to be enriched with missense variants in all three breeds) that has been associate with coat/hair pigmentation in mouse, is associated with meat tenderness in Nellore cattle (Carvalho et al., 2017). A region on chr 12 containing the *CLDN10, DNAJC3, and DZIP1* genes was found to show top 1% overall SNP density as well top 1% novel SNP density in both AFR and DRA. *DNAJC3* is a heat shock protein gene previously associated with embryonic development in Zebra fish (Soares et al., 2012). A region on chr 28 enriched in LoF variants in DRA and NGI contains the *TTC13* gene that has been associated with domestication – adaptation in cattle (Chen N. et al., 2018). Similarly, the *BTBD17* gene in Chr 9 (corresponding region shows enrichment for missense variants in all the three breeds) has been associated with network of genes that regulate milk yield in Holstein cattle (Chen Z. et al., 2018), and also responsible for abortion – embryonic lethalty in dogs. *PAG5* gene in Chr 29 (corresponding region showing non-synonymous variants enrichment only in NGI) have been associated with pregnancy (formation of placenta) in cattle while *R8I2* in Chr 15 have been associated with taste in rat, as well as sensory perception of smell in humans (Ignatieva et al., 2014; Wallace et al., 2015).

### Identification of Selective Sweeps

**FIGURE 4:**
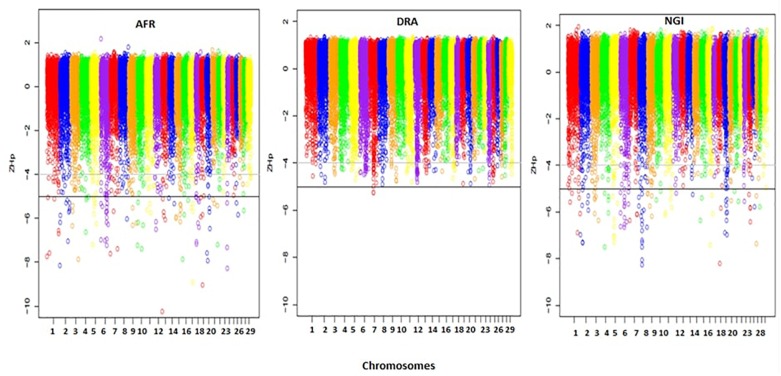
Distribution of ZHp Z-scores across all 29 autosomes for Afrikaner (AFR), Drakensberger (DRA), and Nguni (NGI). The horizontal lines indicate ZHp Z-score thresholds of -4 and -5 used to define candidate and putative selective sweep regions in this study.

A total of 33,467 150 kb sliding windows were used to calculate the Z-transformed pooled heterozygosity (ZHp) scores to identify putative selective sweep regions. The ZHp Z-scores ranged from -10.26 to 2.18, from -5.27 to 1.37, and from -8.27 to 1.94 in AFR, DRA, and NGI, respectively. Thus, there appeared to be regions of excess homozygosity but not excess heterozygosity in the genomes of these animals. Figure 4 show the distributions of the ZHp Z-scores genome-wide for the three indigenous SA breeds. The most noteworthy regions of homozygosity were observed in a region spanning <50 Mb on chromosomes 8 and 19 in NGI, chromosomes 13 and 19 in AFR, and on chromosome 7 in DRA.

The genome-wide screening of the breeds revealed 113 distinct loci with ZHp Z-scores ≤-5, and 157 loci with ZHp Z-scores ≤-4 in AFR, 2 and 152, respectively in DRA, and 108 and 156, respectively in NGI. In total, 465 candidate selective sweeps with ZHp Z-scores ≤-4 were identified across the genomes of AFR, DRA, and NGI and 223 regions were identified as putative selective sweeps (ZHp Z-scores ≤ -5). The lowest number of putative selective sweeps was observed in DRA. The regions identified as candidate selective sweeps (ZHp Z-scores ≤-4) in DRA were located on 26 different chromosomes. We also identified 93 selective sweep regions with extremely low ZHp Z-scores (ZHp-scores ≤-6) as indicated in Table 6.

**Table 6:** List of genes within SNP enriched genomic regions in the top 100 kb window.

Gene	CHR	Function	Species	Reference
**Afrikaner**				
*MOV10*	3	Gene silencing by miRNA	Human	Goodier et al., 2012
*MPV17*	11	Abnormal coat/hair pigmentation, thin skin, decreased body weight, kidney failure, anemia, hypertension, increased heart rate	Mouse	Weiher et al., 1990; Viscomi et al., 2009
*UCN*	11	Increased anxiety, feeding behavior, heart failure, decreased drinking behavior, parkinsonian disorders	Mouse, rat	Vetter et al., 2002
*TRIM54*	11	Premature death, abnormal heart morphology	Mouse	Hwang et al., 2010
*DNAJC5G*	11	Cardiovascular system phenotype, decreased anxiety-related response	Mouse	Rovelet-Lecrux et al., 2012
*WNT4*	2	Serkal syndrome, female sex determination, kidney failure, male sex differentiation	Mammals, mouse	Vainio et al., 1999; Brisken et al., 2000
*CDC42*	2	Negative regulation of gene expression, hair follicle placode formation, spinal cord injuries, bipolar disorder, epilepsy arthritis	Mouse, rat	Erschbamer et al., 2005; Park et al., 2009
*MLANA*	8	Diluted coat color, hair morphology	Mouse	Steingrimsson et al., 2006
*KIAA1549*	4	Decreased total body fat amount, pilocytic astrocytoma (brain tumor)	Human, mouse	Hughes, 1998; Antonelli et al., 2015
*HECTD3*	3	Decreased lean body mass, length, increased total body fat amount	Mouse	Zhang et al., 2009
**Drakensberger**				
*YTHDC2*	10	Prostatic neoplasms	Rat	Arambula et al., 2016
*DCLRE1B*	3	Decreased embryo size, neonatal lethality, cell cycle checkpoint	Mouse, human	Liu et al., 2009; Dronkert et al., 2000
*AP4B1*	3	Spastic paraplegia, autosomal recessive	Mouse, human	Tuysuz et al., 2014
*PTPN22*	3	Autoimmune diseases, enlarged spleen, diabetes mellitus, insulin-dependent	Human, mouse, rat	Bottini et al., 2006; Michou et al., 2007
*ZC3HAV1*	4	Suppression by virus of host molecular function, endosome to lysosome transport	Mouse	Lee et al., 2009
*PSMB11*	10	Increased T-cell proliferation, abnormal self-tolerance	Mouse	Anderson and Takahama, 2012
*AJUBA*	10	Gene silencing by miRNA, wound healing, spreading of epidermal cells, heart contraction, decreased rate, abnormal cell migration	Human, zebrafish, mouse	Bergantinos et al., 2010; Wilkinson et al., 2014
*SLC7A8*	10	Decreased susceptibility to pharmacologically induced seizures	Mouse	Dai et al., 2007
*IFT74*	8	Abnormal lung lobe morphology, notch signaling involved in heart development, cilium assembly	Human, mouse	Kwong et al., 2007; Bhogaraju et al., 2013
*SUPT7L*	11	Abnormal hair texture, decreased body weight, embryonic lethality	Mouse	Bardot et al., 2016
**Nguni**				
*RAB33B*	17	Skeletal system morphogenesis	Human	Bonafe et al., 2015
*SYT10*	5	Shortened circadian period (sleep disorder), sensory perception of smell	Mouse	Anda et al., 2016
*STT3B*	22	Congenital disorder of glycosylation	Human	Scott et al., 2014
*CEACAM16*	18	Deafness, autosomal dominant 4b	Human, mouse	Zheng et al., 2011; Lukashkin et al., 2012
*SRGAP2*	16	Dendritic spine development	Mouse	Charrier et al., 2012
*TMEM98*	19	Nanophthalmia, hemorrhage	Human, mouse	Liao et al., 2016
*CCL17*	18	Staphylococcal pneumonia, bronchiolitis obliterans	Mouse	Montgomery and Daum, 2009
*TXN*	8	Fatty liver, myocarditis, diabetes mellitus	Rat	Chung et al., 2011
*COG5*	4	Congenital disorder	Human	Wu et al., 2004
*AIRE*	1	Reduced fertility, thyroid, and eye inflammation	Mouse	Schaller et al., 2008

A locus with extremely low ZHp Z-score of -10.26 was found in AFR on chromosome 13, but no annotated genes were identified in this region. A protein coding gene, family with sequence similarity 101, member B (*FAM101B*) was identified in a sweep region with a ZHp Z-score of -9.05 in AFR on chromosome 19, and was also found in NGI with a ZHp Z-score of -8.2. This gene is involved in the regulation of the perinuclear actin network and nuclear shape through interaction with filamins, and plays an essential role in the formation of cartilaginous skeletal elements in human. There were also a few overlapping common genes that were identified across all the three breeds that could have been associated with breed formation in cattle. The *KIT* and *MITF* genes on chromosomes 6 and 12 respectively, have been associated with pigmentation in cattle, *KDR* on chromosome 6 is a tyrosine kinase receptor, *ERBB4* on chromosome 2 is associated with a signaling pathway involved in the development and progression of melanocytes in human (Choi et al., 2010). Other genes include *CACNA1C* on BTA5, *LAMC3* on BTA11, *TAS2R16* on BTA4, *UNC93A* on BTA9, *TNFRSF9* on BTA16, *CAV2* on BTA4, and *DCST1* on BTA3. These genes have previously been identified in selective sweep regions in cattle and have been associated with: (1) major depression, (2) the development of brain cortex and formation of axons, (3) dietary habits, (4) associated with Herpes simplex encephalitis type 1, (5) induced by lymphocyte activation, (6) involved in Cystic Fibrosis, and (7) implicated in Down syndrome, respectively (Qanbari et al., 2014). The keratin genes *KRT24, KRT25, KRT26, KRT27*, and *KRT28*; and the heat shock protein gene *HSPB9* found on chromosome 19, which have previously been associated with adaptation to tropical environment in Zebu cattle, were detected in selective sweep regions common to all three breeds. Other associated genes including *ATP2B*, *FMOD*, *WNT5B*, and *PRELP* on chromosome 16, have also previously been identified as being under positive selection in cattle, and were located in sweep regions shared across the three breeds.

## Discussion

Sequencing of individuals can identify millions of SNPs that differ between any two individual genomes (Bischoff et al., 2008), while good coverage ensures better identification of sequencing errors, increasing sequencing accuracy (Borisevich et al., 2017). Pools of DNA were sequenced for Afrikaner, Drakensberger and Nguni to discover new SNPs. The average sequence depth of 21-fold was obtained in this study, a coverage efficient to make accurate SNP calls (Wang et al., 2011). The sequence depth was relative to studies by Eck et al. (2009) and Stothard et al. (2011), but higher than the study by Choi et al. (2015) with an average coverage of 10.71X for Hanwoo and Yanbian cattle, and lower than the 27X mean coverage obtained by Das et al. (2015) for Danish Holstein dairy cattle. The Ti/Vi ratio are computed for quality control of sequence data and are helpful for understanding patterns of DNA sequence evolution (Wang et al., 2014). The quality of sequence data obtained from this study was comparable to the studies of Gayal, Red Angus, and Japanese Black cattle where the Ti/Tv values were 2.32, 2.17, and 2.18, respectively (Mei et al., 2016). These results suggest that the majority of SNPs identified in this study were accurately identified (Choi et al., 2015).

Due to the history of human migration and trading, it is expected that indigenous breeds will often have multiple genetic signatures of origin and admixture, and this has been confirmed by analyses using available molecular data (Hanotte and Jianlin, 2006; Decker et al., 2014; Makina et al., 2014). These analyses have suggested that several ancestral lineages have contributed to today’s genetic pool of livestock (Hanotte et al., 2000; Qi, 2004). The proportion of novel SNPs found in this study was low compared to what was reported by Choi et al. (2013) where 29.4% of SNPs were found to be novel in Korean Black Cattle when compared to the dbSNP version 137. This likely reflects the large number of SNPs that have now been discovered in 1000 Bull Genomes Project (80 million SNPs in Run 6). This shows the effort made by researchers to address the issue of SNP biasness reflected on the current SNP assays of which mostly European breeds were used in the design of these assays. The availability of this data will allow for imputation of genetic variants for genomic prediction and genome wide association studies in all cattle breeds.

Higher number of novel SNPs identified in the study of Mei et al. (2016) could reflect different SNP filtering criteria, the comparison set used (dbSNP Build 140), and the increased divergence of Gayal cattle from the reference genome, Hereford, relative to the SA breeds. The detected SNPs were validated using dbSNP Build 140, which also represents a smaller validation set than was used in this study. The greater number of novel SNPs found in AFR and DRA cattle likely reflects the extent of genetic diversity that exists between these breeds and also their phylogenetic distance from the reference genome. Novel variants characterize the extent of genetic differentiation that exists between individuals and populations (Choudhury et al., 2014). The lower number of novel SNPs found in DRA suggests that the breed might be more closely related to European breeds than the AFR or NGI (Zwane et al., 2016). The complex origins of cattle are associated with both natural and artificial selection, which gave rise to numerous different breeds displaying a broad spectrum of phenotypes. This happened after the global partitioning of the world-wide cattle genetic diversity by three distinct events, two of which involved domestication, and that resulted in European taurines, West African taurines and Zebu from India spreading all over the world through the migration of different tribes (Gautier et al., 2010; Decker et al., 2014).

The identification of functional variants such as missense variants, and variants within upstream and downstream genic regions in indigenous SA cattle will enable the testing of these variants for their effects on complex traits (Koufariotis et al., 2014). While the roles of variation in overlapping genes is less clear, studies have suggested that this could be a mechanism allowing the regulation of key genes in eukaryotes (Park et al., 2009). Further studies of overlapping genes will enable an understanding of the tissue- and developmental-stage regulation of each strand and will provide insight into their mechanisms of evolution (Nakayama et al., 2006). Genetic variants such as insertions, deletions and structural variants may also be applied in association studies and in genomic prediction (Koufariotis et al., 2014).

The number of SNPs that were located on the splice sites were higher than found by Stothard et al. (2011) in Holstein and Black Angus, and Choi et al. (2013) in Heugu cattle. This could be due to the number of samples, breeds used for sequencing and different genetic backgrounds. The number of functional and non-functional genes differs depending on the breeds and the method used for annotation (Das et al., 2015). The number of functionally annotated Indels was slightly higher than the number of detected Indel loci, because a SNP or Indel locus may have multiple annotations (Choi et al., 2015). The numbers of SNPs and Indels identified in this study were slightly greater in NGI and AFR than in DRA due to the higher indicine percentage present in their genomes (Makina et al., 2016). The numbers of nsSNPs segregating in these breeds were greater than for Danish Jutland Cattle, which had 34,257 nonsynonymous substitutions (34,183 missense and 74 initiator codon variants) identified from four cows (Das et al., 2015). The ability to identify non-neutral substitutions could help targeting diseases caused by detrimental mutations, and SNPs that increase the fitness of particular phenotypes (Bromberg and Rost, 2007). In human, among all types of variants, nsSNPs are believed to be the major contributors to heritable diseases. They constitute more than half of the disease-causing genetic changes deposited in the Human Gene Mutation Database (HGMD) (Stenson et al., 2009).

The analysis of SNP density distribution of the four different categories of SNPs was able to identify genomic regions showing distinctive trends among the breeds. Genes showing high densities of different SNP categories were identified to be associated to phenotypes (coat color), adaptation, fertility (embryonic development, placenta formation), production (meat tenderness) and diseases (abortion, induced viral infection), which represent some of the desired traits in livestock production. More validation of these associations are still needed, especially in farm animals. The global study of the 1000 Bulls Genomes Project has mined genes that influence complex genetic traits in cattle, opening the door for researchers to use the same approach to map high-value traits including those important for beef and milk production. We expect the current data would serve as an important resource, for a comprehensive analysis comparing genomic distribution and densities of SNPs on a global scale.

It has been suggested that rare or low-frequency variants may explain a substantial proportion of the heritability of many complex diseases, most of which have previously not been fully captured in GWAS studies (Bang et al., 2014). The power to identify variants associated with traits, particularly those of small effect, could be increased if certain regions of the genome are known to be enriched for trait associations (Koufariotis et al., 2014). However, given the typical genetic architecture of complex traits, such regions are likely to be very few as also observed in this study. Variants in regions of the genome for which the sequence is strongly conserved across species have been proposed as an important annotation class for prioritization since they are potentially regulatory. The majority of these variants are found in non-coding regions, and it is believed that at least some of these are cis regulators for genes (Knight et al., 2011).

Searching for genomic regions of reduced variability as signatures of strong positive selection can also help in identifying causal mutations controlling selected phenotypes (Voight et al., 2006). Selective sweeps and their associated genes provide an insight into the genomic footprints left by natural and artificial selection in indigenous SA breeds. While identifying a selective sweep in the same region in different breeds provides support that a particular genomic region has undergone selection for a given trait, many selection signatures appear to be breed-specific (Gutiérrez-Gil1 et al., 2015). This study identified 465 candidate selective sweeps with ZHp scores ≤ -4 on 29 chromosomes and 223 regions were identified as putative selective sweeps (ZHp Z-scores ≤ -5) on 17 chromosomes. Using BovineSNP50 data, Ramey et al. (2013) identified 28 genomic regions on 15 chromosomes as putatively harboring selective sweeps in 14 breeds. They also identified 85 putative selective sweep regions from 200 to 846 kb in size using the very high density AFFXB1P assay. Only 11 regions were validated as putative selective sweeps using both assays and no selective sweeps overlapped between the taurine and indicine breeds. For several of the detected sweep regions, Ramey et al. (2013) were able to identify the phenotypes and genes that were likely subjected to selection. However, for many of these regions, the selected genes and phenotypes were unclear. But when using NGS, Qanbari et al. (2014) identified 146 regions of positive selection in non-overlapping 40 kb windows across the genome. They were able to localize regions/genes harboring phenotypic characteristics such as patterned pigmentation, brain development and neurobehavioral functioning, sensory perception, immune system, genetic disorders, and blood coagulation. This shows that the amount of data used and the analytical method employed both impact the identification of the number of regions of positive selection. In this study, the number of identified selective sweeps was even higher. A total of 93 putative selective sweeps with extremely low ZHp Z-scores (ZHp Z-scores ≤ -6.0) were identified across the genomes of Afrikaner and Nguni. This information will help in the discovery of disease resistance alleles and for the inference of the events that molded the genetic structure of these populations. These imprints of historic selection/adaptation episodes left in cattle genomes allow one to interpret modern and ancestral gene origins and modifications (Qanbari et al., 2014).

## Conclusion

The SNPs and Indels identified in this study will serve as useful genetic tools, and as candidates in searches for phenotype-altering DNA differences. Novel SNPs provide an insight into the genomic regions that are unique to each breed. Identification of nsSNPs provides the potential for the detection of genes and variants underlying variation in traits of economic importance in these breeds, in particular environmental adaptation. Genes located in genomic regions that are enriched for variation suggests their potential for selection due to effects on phenotypic characteristics. Identification of selective sweeps provides a broader insight into the events that happened during recent selection events and artificial selection processes that have shaped the livestock genome in SA indigenous cattle breeds. These results provide a framework for further genetic association and QTL fine-mapping studies in indigenous SA cattle.

## Author Contributions

AZ designed the experiments, carried out the analysis, and drafted the manuscript. RS and JH assisted with structuring the methodology, data handling, and data management. AC and MM assisted with statistical analysis. AM, EVM-K, and JT structured scientific content. All authors provided editorial suggestions and revisions, read and approved the manuscript.

## Conflict of Interest Statement

The authors declare that the research was conducted in the absence of any commercial or financial relationships that could be construed as a potential conflict of interest.

## Footnotes

1 www.ensembl.org/
